# Development of a population-based cancer case-control study in southern china

**DOI:** 10.18632/oncotarget.19692

**Published:** 2017-07-29

**Authors:** Weimin Ye, Ellen T. Chang, Zhiwei Liu, Qing Liu, Yonglin Cai, Zhe Zhang, Guomin Chen, Qi-Hong Huang, Shang-Hang Xie, Su-Mei Cao, Jian-Yong Shao, Wei-Hua Jia, Yuming Zheng, Jian Liao, Yufeng Chen, Longde Lin, Liming Liang, Ingemar Ernberg, Thomas L. Vaughan, Guangwu Huang, Yi Zeng, Yi-Xin Zeng, Hans-Olov Adami

**Affiliations:** ^1^ Department of Medical Epidemiology and Biostatistics, Karolinska Institutet, Stockholm, Sweden; ^2^ Exponent, Inc., Health Sciences Practice, Menlo Park, CA, USA; ^3^ Division of Epidemiology, Department of Health Research and Policy, Stanford University School of Medicine, Stanford, CA, USA; ^4^ Department of Cancer Prevention Center, Sun Yat-sen University Cancer Center, Guangzhou, China; ^5^ State Key Laboratory of Oncology in South China, Collaborative Innovation Center for Cancer Medicine, Sun Yat-sen University Cancer Center, Guangzhou, China; ^6^ Department of Clinical Laboratory, Wuzhou Red Cross Hospital, Wuzhou, China; ^7^ Wuzhou Health System Key Laboratory for Nasopharyngeal Carcinoma Etiology and Molecular Mechanism, Wuzhou, China; ^8^ Department of Otolaryngology-Head & Neck Surgery, First Affiliated Hospital of Guangxi Medical University, Nanning, China; ^9^ Key Laboratory of High-Incidence-Tumor Prevention & Treatment (Guangxi Medical University), Ministry of Education, Nanning, China; ^10^ State Key Laboratory for Infectious Diseases Prevention and Control, Institute for Viral Disease Control and Prevention, Chinese Center for Disease Control and Prevention, Beijing, China; ^11^ Sihui Cancer Institute, Sihui, China; ^12^ Cangwu Institute for Nasopharyngeal Carcinoma Control and Prevention, Wuzhou, China; ^13^ Department of Epidemiology, Harvard TH Chan School of Public Health, Boston, MA, USA; ^14^ Department of Biostatistics, Harvard TH Chan School of Public Health, Boston, MA, USA; ^15^ Department of Microbiology, Tumor and Cell Biology, Karolinska Institutet, Stockholm, Sweden; ^16^ Public Health Sciences Division, Fred Hutchinson Cancer Research Center, Seattle, WA, USA; ^17^ Department of Epidemiology, University of Washington, Seattle, WA, USA; ^18^ Beijing Hospital, Beijing, China

**Keywords:** case-control study, nasopharyngeal carcinoma, china

## Abstract

With its population of over 1.3 billion persons, China offers abundant opportunities to discover causes of disease. However, few rigorous population-based case-control studies have as yet been conducted in mainland China. We conducted a population-based case-control study of nasopharyngeal carcinoma in Guangdong Province and Guangxi Autonomous Region. We collected questionnaires and biospecimens from incident cases recruited between March 2010 and December 2013, and population-based controls between November 2010 and November 2014. Preparatory activities prior to subject enrollment required approximately 18 months. We enrolled a total of 2554 NPC cases and 2648 controls. Among all identified cases, 83.8% participated. For the participating cases, the median time between diagnosis and interview was 2 days. Among all contacted controls, 82.7% participated. From the enrolled cases, we collected 2518 blood specimens (provided by 98.6% of eligible cases), 2350 saliva specimens (92.0%), 2514 hair specimens (98.4%), and 2507 toenail/fingernail specimens (98.2%). From the enrolled controls, we collected 2416 blood specimens (91.2%), 2505 saliva specimens (94.6%), 2517 hair specimens (95.1%), and 2514 toenail/fingernail specimens (94.9%). We demonstrate that population-based epidemiologic research can successfully be conducted in southern China. The study protocols, databases, and biobank will serve as an extraordinarily valuable resource for testing future etiologic hypotheses.

## INTRODUCTION

The revolution in genomic technologies now offers novel opportunities to discover not only how genetic factors influence risk of disease, but also how these factors interact with environmental and lifestyle factors in the causal web of human diseases. Regardless of how cutting-edge the genomic technologies are, a valid assessment of non-genetic risk factors requires a rigorous epidemiologic study design. For relatively uncommon diseases, case-control studies are more efficient than cohort studies for identifying risk factors. Ideally, case-control studies should include all incident cases of the disease under study in a defined population during a specified time period. In addition, control subjects without the disease should be sampled in such a way (randomly) that they inform about the distribution of exposures in the person-time that gave rise to the cases. In the majority of settings around the globe, these fundamental requirements are difficult or impossible to accommodate. As a corollary, much epidemiologic research is undertaken with suboptimal methods. Even worse, many important human diseases escape proper investigation of their causes altogether.

With its population of over 1.3 billion persons, China offers fertile ground to discover causes of various human diseases. Some or many of these causes or diseases may be unique to China; that is, they may be linked to risk factors that are specific to the Chinese population (such as certain human leukocyte antigen alleles), they may be related to the unprecedented ongoing transition to western lifestyle in this part of the world, or they may pertain to diseases that are rare in other populations. Conversely, knowledge regarding other risk factors and diseases in China may be broadly generalizable to other populations and geographic regions. However, few strictly population-based case-control studies have as yet been conducted in mainland China [1, 2], thereby limiting the extent of epidemiologic findings that can be translated into effective regional and global public health actions.

Nasopharyngeal carcinoma (NPC) is a salient example of a disease that is endemic in southern China and parts of Southeast Asia, but rare in most other parts of the world [3–5]. Nevertheless, there has been little progress in understanding the complex multifactorial etiology of this enigmatic malignancy during the last several decades, due in part to the shortage of high-quality epidemiologic studies. Therefore, we undertook a large, population-based case-control study of NPC in southern China (the NPC Genes, Environment, and EBV (NPCGEE) study) with the specific aims of understanding the independent and interactive roles of genes, environmental exposures, and infection with the Epstein-Barr virus (EBV) in disease etiology. In the context of this study, we were able to develop and evaluate study design strategies aimed to improve the validity and generalizability of our findings. We collected detailed in-person survey data and established a large repository of biological specimens that will enable a multitude of molecular and genetic investigations. Because these methods may be applicable to many other diseases in this geographic region, our intent in this paper is to report the study methods in detail in the hope that this example will encourage scientists to undertake similar studies of other disease phenotypes in the Chinese population.

## RESULTS

**Figure 1:**
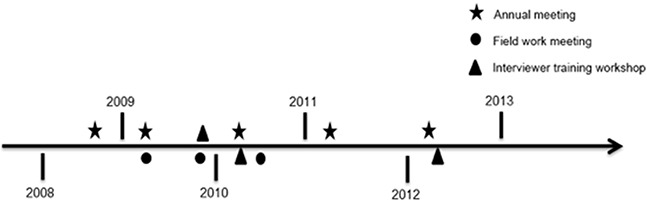
Timeline for meetings and training workshops Schematic showing the timeline of annual research meetings, field work meetings, and interviewer training workshops held between 2008 and 2013.

Preparatory activities prior to subject enrollment required approximately 18 months. This extended period was necessitated by the absence of pre-existing infrastructure for the conduct of population-based epidemiologic research in southern China. Activities during this period included developing protocols for study recruitment and participation, designing the study questionnaires and databases, establishing biospecimen collection and storage protocols, selecting biological assays, hiring and training project staff, purchasing major study equipment, forming contact networks with regional hospitals to facilitate rapid identification and recruitment of incident NPC cases, and obtaining institutional review board approval for human subjects research. To further build research infrastructure in the study area, we also conducted an introductory course in epidemiologic methods at the Sun Yat-Sen University Cancer Center in Guangzhou. Before and during the fieldwork phase we also held three instructional workshops for interviewer training (Figure 1).

**Figure 2:**
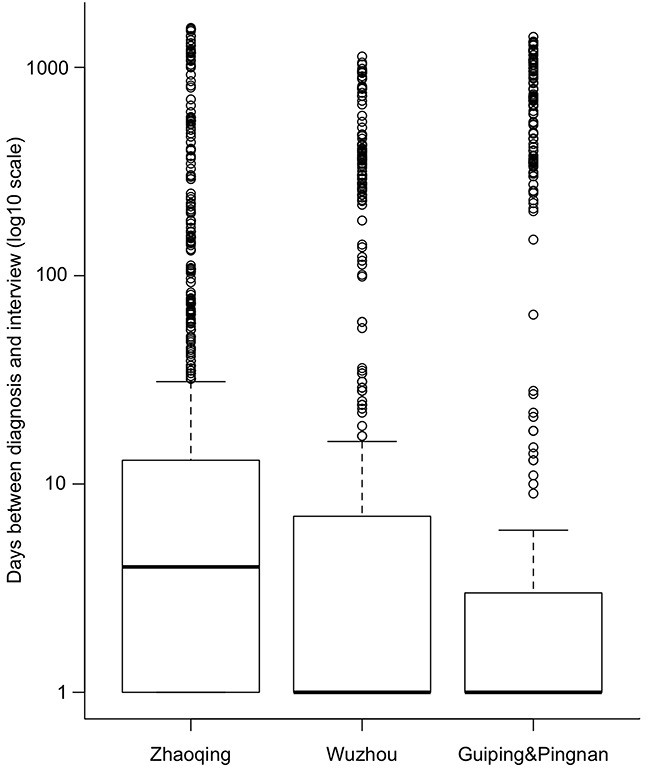
Box plot of days (in log10 scale) between diagnosis and interview by residential area among cases The day of zero was set as one in log scale.

By the end of the study recruitment phase, which ended in December 2014, we enrolled a total of 2554 NPC cases and 2648 population-based controls (Tables 1 and 2). Based on historical incidence rates of NPC, the anticipated number of eligible cases in the study area was 2975. Through our rapid case ascertainment system, we identified a total of 3047 incident cases during the study period. Thus, the participation rate calculated based on the number of expected cases was 85.8%, and the rate calculated based on the number of identified cases was 83.8%. Among the participating cases, the median time between diagnosis and interview was 2 days (interquartile range: 0 to 10 days). There was no variation in the median time between diagnosis and interview by sex (*P*=0.33) or age group (in 10-year categories, *P*=0.89), albeit the median day was slightly higher in Zhaoqing than that in Wuzhou or Guiping/Pingnan (*P*<0.001, Figure 2).

**Table 1:** Numbers and proportions of nasopharyngeal carcinoma (NPC) cases expected, identified, and enrolled in Guangdong Province and Guangxi Autonomous Region, China, 2010-2013

Residential area	No. expected	No. identified	No. enrolled	% Enrolled/expected	% Enrolled/identified
Zhaoqing					
Urban (Zhaoqing city)	196	270	243	124.0%	90.0%
Rural	1309	1258	1063	81.2%	84.5%
Total	1505	1528	1306	86.8%	85.5%
Wuzhou					
Urban (Wuzhou city)	126	178	146	115.9%	82.0%
Rural	609	614	543	89.2%	88.4%
Total	735	792	689	93.7%	87.0%
Guiping & Pingnan					
Urban (Downtown)	72	79	60	83.3%	75.9%
Rural	663	648	499	75.1%	76.9%
Total	735	727	559	75.9%	76.8%

**Table 2:** Numbers and proportions of population controls selected, enrolled, and not enrolled (by reason) in Guangdong Province and Guangxi Autonomous Region, China, 2010-2014

	Urban	Rural	Total
No. selected	546	3386	3932
No. enrolled, interviewed in person	294	2231	2525
No. enrolled, interviewed by phone	0	123	123
No. emigrated	24	114	138
No. not contacted	116	614	730
No. with outdated contact information	61	110	171
No. with a history of working outside of the study area for > 10 years	55	504	559
No. ill or deceased	16	74	90
No. refused	96	230	326
% Enrolled/contacted (range by study center)	68.4% (65.8-69.0%)	84.9% (83.5-87.0%)	82.7% (81.7-84.9%)

A total of 3932 potentially eligible controls were randomly selected for study recruitment (Table 2 and details by residential areas are shown in Supplementary Table 1). Of these individuals, we were able to contact 3202 (81.4%). The overall participation rate among contacted potential controls was 82.7%. The participation rate was 84.9% for rural controls and 68.4% for urban controls.

Data were misplaced for one case and 17 controls, and 6 controls were not within the eligible age range at the time of the interview. Thus, we included 2553 cases and 2625 controls in the final analysis. The distribution of baseline demographics of cases and controls is shown in Table 3. Cases were slightly younger than controls, a difference that is attributable mainly to the fact that we interviewed controls on average approximately one year after the date of selection, as well as a somewhat lower control participation rate in younger age groups (data not shown). Cases were also more likely to be less educated and to be Cantonese than controls.

**Table 3:** Characteristics of nasopharyngeal carcinoma (NPC) cases and controls, Guangdong Province and Guangxi Autonomous Region, China, 2010-2014

Characteristics	Cases (n=2553)		Controls (n=2625)		*P* value
	n	%	n	%	
**Residential area**					0.31
Zhaoqing	1305	51.1	1347	51.3	
Wuzhou	689	27.0	667	25.4	
Guiping/Pingnan	559	21.9	611	23.3	
**Gender**					0.82
Male	1874	73.4	1934	73.7	
Female	679	26.6	691	26.3	
**Age at diagnosis/referral (years)**					
Mean (SD) ^*^	48.6 (10.7)		49.7 (10.9)		<0.001
20-29	87	3.4	82	3.1	0.01
30-39	425	16.6	377	14.4	
40-49	918	36.0	901	34.3	
50-59	691	27.1	735	28.0	
60-74	432	16.9	530	20.2	
**Education level (years)**					0.004
≤ 6	1013	39.7	943	35.9	
7- 9	1023	40.1	1055	40.2	
10-12	410	16.1	486	18.5	
> 12	107	4.2	141	5.4	
**Marital status**					0.26
Unmarried	146	5.7	170	6.5	
Married	2407	94.3	2455	93.5	
**Ethnicity**					0.81
Hans	2507	98.2	2580	98.3	
Others	46	1.8	45	1.7	
**Dialect**					<0.001
Cantonese	2496	97.8	2535	96.6	
Hakka	51	2.0	51	1.9	
Others	6	0.2	39	1.5	

SD = standard deviation.

^*^P value was determined by a two-sided t-test. Other *P* values were determined by a chi-square test.

Table 4 shows the proportions of NPC cases and population controls who provided study biospecimens. From the enrolled cases, we collected 2518 blood specimens (provided by 98.6% of eligible cases), 2350 saliva specimens (92.0%), 2514 hair specimens (98.4%), and 2507 toenail or fingernail specimens (98.2%). Overall, 92% of cases provided biospecimens prior to initiating treatment for NPC. From the enrolled controls, we collected 2416 blood specimens (91.2%), 2505 saliva specimens (94.6%), 2517 hair specimens (95.1%), and 2514 toenail or fingernail specimens (94.9%).

**Table 4:** Proportions of nasopharyngeal carcinoma (NPC) cases and population controls who provided study biospecimens, Guangdong Province and Guangxi Autonomous Region, China, 2010-2014

NPC cases					
Area	No. enrolled	No. with blood (%)	No. with saliva^*^ (%)	No. with hair (%)	No. with toenail or fingernail (%)
Zhaoqing	1306 (100)	1277 (97.8)	1209 (92.6)	1274 (97.5)	1282 (98.2)
Wuzhou	689 (100)	689 (100.0)	600 (87.1)	686 (99.6)	671 (97.4)
Guiping & Pingnan	559 (100)	552 (98.7)	541 (96.8)	554 (99.1)	554 (99.1)
Total	2554 (100)	2518 (98.6)	2350 (92.0)	2514 (98.4)	2507 (98.2)
**Population controls**					
**Area**	**No. enrolled**	**No. with blood (%)**	**No. with saliva (%)**	**No. with hair (%)**	**No. of toenail or fingernail (%)**
Zhaoqing	1356 (100)	1132 (83.5)	1213 (89.5)	1233 (90.9)	1237 (91.2)
Wuzhou	680 (100)	680 (100.0)	680 (100.0)	680 (100.0)	680 (100.0)
Guiping & Pingnan	612 (100)	604 (98.7)	612 (100.0)	604 (98.7)	597 (97.5)
Total	2648 (100)	2416 (91.2)	2505 (94.6)	2517 (95.1)	2514 (94.9)

^*^ Saliva sample collection started in July 2010, whereas case enrollment began in March 2010.

## DISCUSSION

This first rigorous population-based molecular case-control study of NPC in southern China demonstrates that this highly informative type of epidemiologic research can be conducted successfully in a region with boundless possibilities to inform our understanding of the causes and prevention of cancer and other chronic diseases. Perhaps the two most challenging aspects of conducting this study were ensuring complete case ascertainment and unbiased control selection. These steps, which are prerequisites of any valid population-based case-control study, required many months of planning, communication, and collaboration.

Selection of an appropriate geographic setting is essential for a successful epidemiologic study. For this NPC case-control study, we chose an area with a relatively high incidence of NPC and a relatively low rate of residential mobility. Even within China, different regions and ethnic groups experience substantial variation in NPC risk, with rates generally increasing from northern to southern China, and peaking in southern Cantonese populations [6]. Thus, this study was based in the area with the world's highest incidence rates of NPC.

Our selection of the 13 eligible cities/counties also took into account the fact that residents of other parts of Guangdong and Guangxi are more geographically mobile due to a more highly developed economy in those areas. We found that 14.2% of selected potential controls (559 out of 3932) could not be contacted due to having a history of working outside of the study area for > 10 years; these individuals comprised the majority of non-contacted potential controls (75.8% of 730). The relatively low proportion of individuals with long-term work outside of the study area allayed our initial concerns that rapid economic growth in China would have led to high mobility of local residents in the study region. The relative geographic stability of the populations in the 13 selected areas made residents of these cities/counties easier to identify and contact, thereby improving the probability of our enrolling a representative study population.

Inconsistent results regarding associations of environmental risk factors with NPC risk in prior studies [3] may be due in part due to a shortage of studies with a strictly population-based design. Therefore, we aimed in this study to assess environmental exposures (particularly with regard to diet, smoking, and medical history) in a control population that was representative of the general, non-hospitalized population that would have been identified through the rapid case ascertainment system if they had developed NPC. In addition, to minimize information bias, we devoted extensive efforts to questionnaire development and interviewer training. We used an electronic structured questionnaire to conduct primarily face-to-face, audiotaped interviews with study participants. Questionnaire data were automatically flagged for logic errors and missing values, and mistakes were corrected by making comparisons against audio recordings or by re-contacting participants. We analyzed the questionnaire data every 3 months and sent reports to interviewers if any common mistakes were made. We also required each interviewer to interview approximately an equal number of cases and controls and interviewers were required not to treat cases and controls differentially.

Despite the considerable methodological strengths of our study, some limitations merit discussion. First, questionnaire data were retrospectively self-reported and may have been inaccurate, especially for distant past exposures. Recall bias may have occurred if cases remembered or reported their life-style habits differently than controls, despite interviewers’ efforts to treat cases and controls in the same manner. Second, the participation rate was lower among urban than rural controls, potentially introducing a degree of selection bias. In addition, controls were on average one year older than cases, although age differences can be controlled for in multivariate regression analyses. Third, although all NPC cases were histopathologically confirmed, information on specific histological type was not available for some NPC cases. However, undifferentiated nonkeratinizing NPC comprises the vast majority (over 90%) of NPC cases in southern China, while the rest are mostly differentiated nonkeratinizing and rarely squamous cell carcinoma [7]. Therefore, our results are likely to apply mainly to undifferentiated nonkeratinizing NPC. Fourth, the time from biospecimen collection to long-term freezer storage was more than one day for some study subjects. The delay may lead to incorrect estimates of some biomarkers that are sensitive to processing and storage time [8, 9], and bias could be introduced by any differences in processing time between cases and controls. In addition, we could not collect blood samples under fasting status, and not all biospecimens from cases were collected before treatment initiation. Counterbalancing these limitations are the substantial strengths of our study, which include its large size, population-based design, and setting in a relatively culturally and ethnically homogeneous NPC-endemic region.

In summary, we have demonstrated that a rigorous population-based case-control study can be conducted successfully in southern China. The study protocols, databases, and biobank will serve as an extraordinarily valuable resource for testing future hypotheses, and they provide an example that can be followed in future population-based case-control studies of other chronic diseases in this geographic region. In particular, with an increasing recognition of the importance of infectious agents in malignancy, this epidemiologic study of NPC provides an excellent model for investigating gene-virus-environment interactions in the etiology of cancer. This study could produce a quantum leap in the knowledge of the causes and, ultimately, prevention of NPC.

## MATERIALS AND METHODS

### Defining the study base

Disease events occur among humans as time elapses. The fundamental challenge in epidemiologic research is to harvest information on causal relations between exposures and diseases from the pool of accrued person-time. To be informative, the person-time defined for a specific study needs to be large enough to minimize the influence of random variation. In addition, the exposures of interest must be sufficiently prevalent and variable among members of the population to enable useful comparisons. Finally, it must be feasible to enroll representative individuals into the study and collect valid information on their exposures, covariates, and outcomes.

For our case-control study of NPC, we defined the study base as persons living in 13 cities/counties (Deqing, Fengkai, Gaoyao, Huaiji, Sihui, Zhaoqing, Guangning, Wuzhou, Cenxi, Cangwu, Tengxian, Pingnan, and Guiping) in Guangdong Province and Guangxi Autonomous Region in southern China between March 2010 and December 2013 for cases, and between November 2010 and November 2014 for controls. These 13 areas were chosen because of their high incidence of NPC, their geographic contiguity, their existing opportunities for collaboration with local investigators, and their relatively stable population base compared with other, more urban areas in this geographic region. The total population of the 13 study cities/counties is approximately 8 million and the estimated total number of incident NPC cases is approximately 850 per year, based on information from local cancer registries located in Sihui and Cangwu counties [10]. To ensure adequate statistical power to detect interactive risk factors, we initially aimed to recruit 2,600 cases and 2,600 controls.

The study inclusion and exclusion criteria were designed to maximize the validity and generalizability of the results. Eligible subjects were those currently with a residence in the study area, which defined the source population; aged between 20 and 74 years, because NPC is exceedingly rare among children and young adults, NPC incidence declines after approximately age 64 years in China, and elderly adults generally have more comorbidities that preclude study participation; without prior malignant disease or congenital or acquired immune deficiency, because these conditions alter NPC risk and potentially its causes; fluent in Cantonese, in which the study interview was conducted; and mentally and physically competent to participate in the study interview.

### Case enrollment

Timely case recruitment is essential to ensure that patients with advanced disease are not selectively lost, to minimize the probability that behavioral factors are affected by disease diagnosis, and to enable the collection of biospecimens that are not altered by treatment. To this end, we developed a rapid case ascertainment system involving a network of physicians who diagnosed and/or treated NPC at hospitals in the study area. We hired a total of 12 full-time interviewers along with a few part-time interviewers (contact persons), and 3 full-time study coordinators. Most interviewers were retired nurses from the participating hospitals thus they could also help to identify newly diagnosed NPC cases. Contact persons notified the study personnel (3 study coordinators) as soon as a new NPC case was histopathologically confirmed, after which physician permission was sought to contact each patient who was deemed by the physician to be physically and mentally able to participate in the study.

This rapid case ascertainment system was backed up by two population-based cancer registries that have collected cancer incidence data in Sihui County since 1977 and Cangwu County since 1982. However, these registries do not cover the entire population of the study area and often are not immediately notified about new cancer diagnoses, making them insufficient on their own to fulfill the aims of our study. In general, local cancer registries cover only 6% of the national population of China [11]. The lack of a nationwide cancer registry and the scarcity of high-quality local cancer registries make population-based cancer case identification difficult in China, especially in rural regions. Therefore, ad-hoc case ascertainment networks based on local hospitals are virtually required for population-based case identification in most parts of the country.

**Figure 3:**
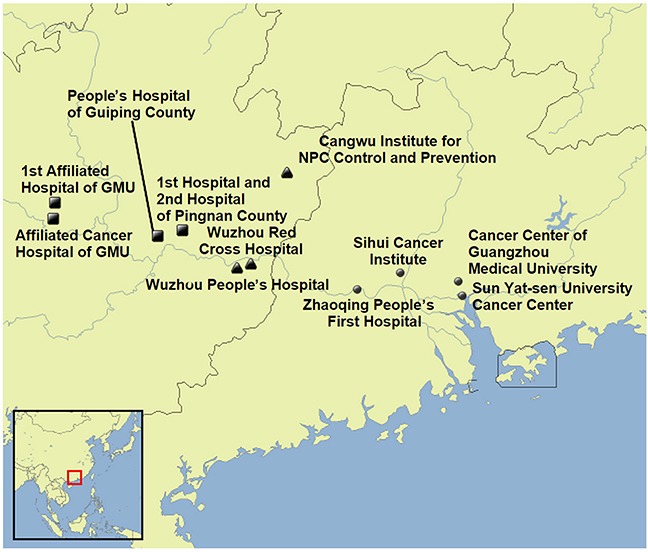
Geographic locations of participating hospitals and cancer research institutions GMU, Guangxi Medical University; NPC, nasopharyngeal carcinoma.

The foremost lesson learned from our experience with rapid case ascertainment in southern China is that such a system requires substantial initial effort to establish strong collaborative relationships with regional hospitals and cancer research institutes where the disease is diagnosed and treated (Figure 3). Building this contact network required approximately 1 year prior to beginning case enrollment, and efforts to maintain and expand the network continued throughout the remaining 3.5 years of the enrollment period. For example, a low case participation rate compared with expected numbers at one study site was attributable largely to the failure of staff at a single hospital to notify study personnel when new NPC cases were diagnosed. Thus, continuous communication with hospital staff and monitoring of participation rates (mainly through our full-time study coordinators) are necessary to ensure complete case ascertainment.

Because NPC treatment often is not initiated immediately after diagnosis, some NPC patients were contacted prior to hospitalization and treatment. First contact was attempted by telephone or, if a telephone number was not available, with a direct home visit based on the address obtained from consultation with town hospitals through the Chinese public health network. Interviews and collection of biospecimens were performed at the town hospitals.

Since not all newly diagnosed NPC cases were treated in the participating hospitals, potentially leading to incomplete case ascertainment, we also contacted NPC patients who were not identified through the rapid case ascertainment system but were reported by town hospitals. Nevertheless, 85.7% (2188 of 2554) of the enrolled cases were interviewed within 30 days after diagnosis, thereby demonstrating the high coverage and efficient turnaround of the rapid case ascertainment system.

### Control enrollment

The case eligibility criteria were applied equally to controls to ensure that both groups arose from the same study base. Random control selection, which is important to ensure that the exposure distribution among enrolled controls is representative of that in the underlying study base, is facilitated in China by the maintenance of a computerized, continuously updated total population registry in each administrative area. Information in these registries includes the name, age, sex, telephone number, and home address of each local resident. Controls were randomly selected every six months from total population registries in the study area, with frequency matching to the expected five-year age and sex distribution of the cases. The 3 study coordinators supervised by the project leader conducted the sampling plan. Study coordinators sent number of controls needed in each age and sex stratified stratum, along with a set of random numbers, to the director of the local Total Population Registry, and the staff there selected controls from the database. We first attempted to contact controls through village doctors in rural areas or community committee members in urban areas. Interviews were conducted at the subject's home or a nearby hospital. Potential control subjects with outdated contact information or a history of working outside of the study area for more than 10 years, as identified with the help of the local government in each town or community, were replaced.

To rule out the possibility that the selected controls were NPC patients, during interview, self-reported cancer diagnosis history was collected and was validated by village doctors, town hospitals, or community health centers, wherever such records were available. In addition, two anti-EBV antibodies (IgA antibodies against viral capsid antigen [VCA] and EBV nuclear antigen 1[EBNA1]) with a high accuracy of NPC diagnosis [12, 13] were tested, and those seropositive controls were referred for fibrotic endoscopy to rule out NPC onset. During the study period, two potential controls were detected with NPC, thus were classified into case group.

A challenge of using total population registries for control identification in China is that the contact information is often outdated, especially in urban areas, where the population tends to be more mobile than in rural areas. Moreover, many adults from rural areas tend to work in large cities outside of their hometown. Thus, although direct refusal rates may be low, overall participation rates are diminished by a high rate of failure to contact potential controls. Low participation rates, in turn, raise the concern that participating controls are non-representative of the underlying exposure person-time. In the early phases of control recruitment into our study, the overall control participation rate was below 60%, conferring a sizeable probability of selection bias. To increase controls’ participation rates, we offered free serological tests for 2 anti-EBV antibodies and sent results back to the participants. In addition, we attempted to contact potential controls just prior to the Chinese Spring Festival, when many people return to their hometown (i.e., their permanent place of residence) to celebrate the holiday. Furthermore, for a small set of control subjects, we performed the interview by telephone after several failed attempts at a face-to-face interview. These recruitment strategies substantially improved control contact and participation rates.

A valuable lesson learned from our experience with control recruitment in southern China is that working with local government officials to identify and contact randomly selected controls can substantially improve the accuracy of contact information and, consequently, increase participation rates. Partnering with the local government is especially important in urban areas to ensure that participating controls are not skewed toward rural populations and others with more stable residential histories.

### Interviews

Questionnaire development required approximately 12 months. This period included a pilot study, conducted in Sihui and Cangwu Counties, that was designed to develop a validated electronic questionnaire for use in the target population. For example, for the development of a food frequency questionnaire, we firstly considered more than 200 types of food typically consumed in southern China. We then identified the most commonly consumed items for inclusion in the pilot questionnaire. By combining the food frequency questionnaire results with findings from published studies in southern China [14–16], we selected 77 food items, which were estimated to cover about 80% of the total energy intake per day in the target population, for inclusion in the final study questionnaire. We also drafted an interviewer manual that described survey techniques, common mistakes during interviews, and pictures of serving sizes for various food items. Potential NPC risk factors, including socioeconomic background, ethnicity, residential history, occupational history, smoking habits, alcohol consumption, herbal medicine use, medical history, reproductive history, family history of cancer, and current and past diet, were assessed in our study using a structured, computerized questionnaire administered to subjects in person by trained interviewers in the hospital or at home. Interviews were audiotaped for quality control, and logic checks and automatic skip patterns were built into the questionnaire. Preliminary questionnaire data were analyzed periodically to ensure that results were within expectation.

Lessons learned are that a stable interviewer team, with periodical interviewer re-training, is important to maintain questionnaire data quality (Table 5). Although interviewers in this study could not be blinded to subjects’ case-control status, we attempted to minimize interviewer bias by requiring interviewers to adhere strictly to the questionnaire format, and to treat all subjects as uniformly as possible. We provided training in the form of a written manual and in-person courses on interviewing technique and the content of the study questionnaire. We also tried to assign each interviewer to approximately equal numbers of cases and control subjects.

**Table 5:** Questionnaire completeness among nasopharyngeal carcinoma (NPC) cases and population controls in Guangdong Province and Guangxi Autonomous Region, China, 2010-2014

Questionnaire topic	Missing among NPC cases (n=2553)^*^		Missing among population controls (n=2631)^*^	
	no.	%	no.	%
Demographics	0	0	0	0
Type of residence	3	0.1	3	0.1
Occupation	9	0.4	9	0.3
Medical history				
Head and neck disease	4	0.2	2	0.1
Oral health	10	0.4	4	0.2
Family history of NPC	6	0.1	3	0.1
Tobacco smoking	6	0.2	5	0.2
Alcohol drinking	12	0.5	17	0.6
Tea drinking	9	0.4	6	0.2
Dietary habits				
2000-2002 or adolescence (ages 16-18 years)	8	0.3	10	0.4
Childhood	7	0.3	7	0.3

### Biobank

From each participant we requested blood for serologic and host genotyping analyses, saliva for oral microflora and EBV genotyping analyses, and hair and toenail/fingernail samples for chemical and trace element analyses. Planning for biobanking required advance development of protocols for the preparation, collection, processing, transportation, and storage of each type of biospecimen. Briefly, blood samples were stored at around 4°C for up to three days and then transported to laboratory. Serum, plasma, red blood cell, and buffy coat were extracted and stored in −80°C. Saliva samples were stored at around 4°C for up to three days and then transported to laboratory and stored in −80°C (−20°C for the Wuzhou site). Hair and toenail/fingernail samples were put into envelopes and stored in room temperature.

While the original project proposal had planned for the transport of biospecimens overseas for molecular and genetic analyses, the rapid development of technology and scientific expertise in China meant that all required capabilities were amply available domestically by the time we began to contemplate specific biomarkers and assay methods for analyses.

Lessons learned are that a barcoded biobanking system following standard operating procedures needs to be planned well before study starts. Experimental protocols need to consider limitations in field work. Although we aimed to pretreat blood samples and transfer biospecimens into freezers on the day of collection, at some remote study locations we were forced to keep samples at around 4°C for up to three days. These storage conditions might influence the ability to detect some biomarkers; therefore, we recorded details of biospecimen collection and processing procedures for each subject. For saliva collection, we adopted a method for convenient collection under field conditions, in which lysis buffer was added in the collection tube before saliva collection, thereby enabling specimens to be stored at room temperature for a few days [17].

### Statistical analyses

Differences in distribution of time between interview and diagnosis among cases were compared by age, sex, and residential area using the Wilcoxon rank-sum test for categorical variables or the Kruskal-Wallis test for continuous variables. We compared differences in demographics between NPC cases and controls by using the chi-squared test for categorical variables and Student's t-test for continuous variables.

### Ethical consideration

This study was approved by institutional/ethics review boards at all participating centers. Written or oral informed consent was obtained from all individual participants included in the study.

## SUPPLEMENTARY MATERIALS TABLE



## Abbreviations

CI: confidence interval
EBV: Epstein-Barr virus
GMU: Guangxi Medical University
NPC: nasopharyngeal carcinoma
NPCGEE: NPC genes, environment, and EBV
