# Supplementary material for: Development of a population-based cancer case-control study in southern china
Source: Oncotarget. 2017 Jul 29;8(50):87073–85. doi: 10.18632/oncotarget.19692 (PMC5675616; doi:10.18632/oncotarget.19692)
Supplement: Supplementary file 1 [file oncotarget-08-87073-s001.pdf]

Development of a population-based cancer case-control study in southern china

SUPPLEMENTARY MATERIALS

Supplementary Table 1: Numbers and proportions of population controls selected, enrolled, and not enrolled (by reason) in Guangdong Province and Guangxi Autonomous Region by residential areas, China, 2010-2014

	Zhaoqing area			Wuzhou area			Guiping & Pingnan area			Total		
	Urban	Rural	Total	Urban	Rural	Total	Urban	Rural	Total	Urban	Rural	Total
# of selected	251	1700	1951	188	737	925	107	949	1056	546	3386	3932
# of face to face interview	140	1093	1233	106	574	680	48	564	612	294	2231	2525
# of phone interview	0	123	123	0	0	0	0	0	0	0	123	123
# of emigration	5	63	68	9	27	36	10	24	34	24	114	138
# of cannot be contacted	48	244	292	34	69	103	34	301	335	116	614	730
# of outdated contact information	12	18	30	32	39	71	17	53	70	61	110	171
# of a history of working outside of the study area for more than 10 years	36	226	262	2	30	32	17	248	265	55	504	559
# of sickness or death	9	50	59	4	9	13	3	15	18	16	74	90
# of refusal	49	127	176	35	58	93	12	45	57	96	230	326
% enrolled/contacted	69.0%	83.5%	81.7%	68.8%	85.9%	82.7%	65.8%	87.0%	84.9%	68.4%	84.9%	82.7%
